# HARNESSING COMPLEMENTARY RESEARCH SKILLS TO ADVANCE RESEARCH ON FINANCIAL DECISION MAKING

**DOI:** 10.1093/geroni/igad104.1240

**Published:** 2023-12-21

**Authors:** Marti DeLiema, Laura Hemmy

**Affiliations:** University of Minnesota, Minneapolis, Minnesota, United States; University of Minnesota, Minneapolis, Minnesota, United States

## Abstract

Financial decision making comprises a set of cognitive skills including problem-solving, critical thinking, and an understanding of key financial facts that help people make informed decisions about their money. Research indicates that financial decision making is one of the earliest areas of cognitive functioning to be affected by aging and by aging-related neurological disease. The Financial Decision Making Special Interest Group (SIG) has brought together several interdisciplinary researchers from social work and neuropsychology to advance our understanding of financial decision making trajectories in older adults, and how these trajectories impact their risk of harmful financial outcomes. Members bring different skills to the collaboration--cognitive assessment, working with clinical populations, and deep knowledge of the effects of poor decision making on financial security and risk of fraud and exploitation. In this presentation, SIG members will share how they overcame challenges to develop a collaborative research agenda, including building membership and identifying samples in which to pilot measures of financial decision making.

